# Allograft Prosthetic Composite (APC) for Proximal Humeral Bone Deficiency in Revision Reverse Shoulder Arthroplasty: A Technical Note and Systematic Review

**DOI:** 10.3390/jcm13206290

**Published:** 2024-10-21

**Authors:** Hean Wu Kang, Christopher Child, Kristine Italia, Mirek Karel, Luke Gilliland, Helen Ingoe, Jashint Maharaj, Sarah Whitehouse, Kenneth Cutbush, Ashish Gupta

**Affiliations:** 1 Queensland Unit for Advanced Shoulder Research (QUASR), Queensland University of Technology (QUT), Brisbane, QLD 4000, Australia; heanwu@hotmail.com (H.W.K.); child@ik.me (C.C.); kristine@akunah.com (K.I.); mirekkarel@outlook.com (M.K.); luke@akunah.com (L.G.); helen.ingoe@otago.ac.nz (H.I.); jashint@qoc.com.au (J.M.); s.whitehouse@qut.edu.au (S.W.); k.cutbush@uq.edu.au (K.C.); 2 Australian Shoulder Research Institute, Brisbane, QLD 4000, Australia; 3 Greenslopes Private Hospital, Brisbane, QLD 4120, Australia; 4 St Andrew’s War Memorial Hospital, Brisbane, QLD 4000, Australia; 5 Akunah, Brisbane, QLD 4101, Australia; 6 School of Medicine, University of Queensland, Brisbane, QLD 4072, Australia

**Keywords:** allograft prosthetic composite, revision shoulder arthroplasty, preoperative planning, proximal humeral bone deficiency

## Abstract

**Background**: Proximal humeral bone deficiency in revision shoulder arthroplasty is an emerging and challenging problem as the use of reverse shoulder arthroplasty (RSA) increases. This paper presents a technical note discussing our detailed preoperative planning steps, surgical techniques, and their rationale in carrying out the use of an allograft prosthetic composite (APC) to address proximal humeral bone deficiency in revision RSA. The outcomes of this technique are also presented. This paper also presents a systematic review to further discuss the existing literature on RSA with APCs. **Methods**: The preoperative surgical planning and the surgical technique employed to execute proximal humeral reconstruction using APC during revision arthroplasty are discussed in the technical note. The preliminary clinical and radiological results of five patients who underwent revision shoulder arthroplasty with proximal humeral reconstruction using APCs are presented. The PRISMA guidelines were followed to perform the systematic review. A systematic search using PubMed, Embase, and Cochrane databases was conducted. All studies involving RSA and APCs were pooled, and the data were extracted and analyzed. **Results**: A total of 14 studies were eligible for inclusion in the systematic review, with a total of 255 patients and a mean follow-up of 57 months. All studies in the systematic review and the patients included in the author’s case series showed improvements in the level of pain, range of motion, function, and satisfaction. Graft incorporation in the systematic review was 84%. **Conclusions**: Based on the available literature and the results of our case series, the use of an APC construct is a viable option for proximal humeral bone deficiency in revision shoulder arthroplasty.

## 1. Introduction

Proximal humeral bone deficiency in shoulder arthroplasty is a very challenging problem. This is often encountered in complex proximal humeral fractures or their sequelae, such as nonunions [1], and in the revision of failed hemiarthroplasties [2]. Other common etiologies include tumor resection [3,4] and the revision of shoulder arthroplasty cases for periprosthetic fractures around shoulder arthroplasty [5,6]. Patients are often elderly with poor bone quality. Osteopenia coupled with relatively large humeral stems resulting in high filling ratios can lead to proximal stress shielding and subsequent bone loss during revision surgery [7].

In revision surgery, achieving a stable construct to allow for suitable shoulder function postoperatively is paramount. This is accomplished by adequately restoring the humeral length and height, with associated soft-tissue reconstruction and tensioning [8,9]. Failure to recognize humeral deficiency and address humeral height poses significant problems, as it leads to inadequate tension and subsequent instability. Usually, the surgeon is left to rely on a thicker polyethylene construct and extra deltoid tensioning for stability. The inability to reattach or reconstruct remaining rotator cuff tendons and other supporting soft tissues also contributes to instability and poor outcomes. As such, rigorous preoperative planning to ascertain pre-morbid humeral dimensions to ensure appropriate humeral length and height restoration with the re-establishment of proper soft-tissue tension is of the utmost importance [10]. This paper presents a technical note to discuss the detailed preoperative planning steps, surgical techniques, and their rationale in carrying out the use of an allograft prosthetic composite (APC) to address proximal humeral bone deficiency in revision reverse shoulder arthroplasty (RSA). The clinical and radiological outcomes of this technique are also presented. This paper also provides an updated overview of the current literature through a systematic review.

## 2. Approach to Performing RSA with APC: Preoperative Planning

### 2.1. Assessment of Pre-Morbid Humeral Length and Shape

**Figure 1:**
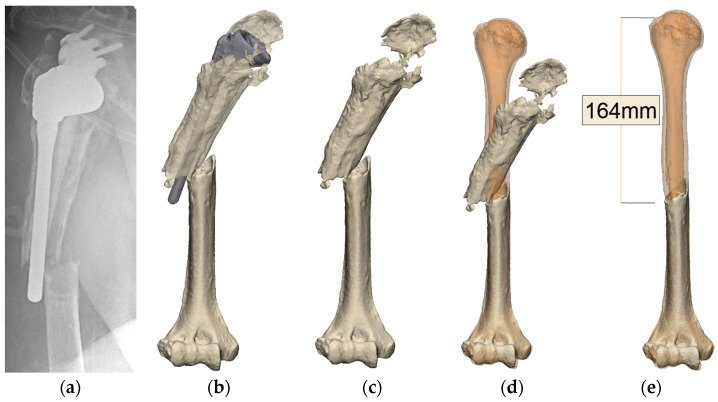
(**a**) Preoperative X-ray of right humerus with periprosthetic fracture; (**b**) 3D modeling with the implants in situ; (**c**) metal subtraction of the right humerus; (**d**) mirroring of the contralateral humerus (orange); (**e**) extent of defect determined after overlaying the contralateral humerus (orange).

**Figure 2:**
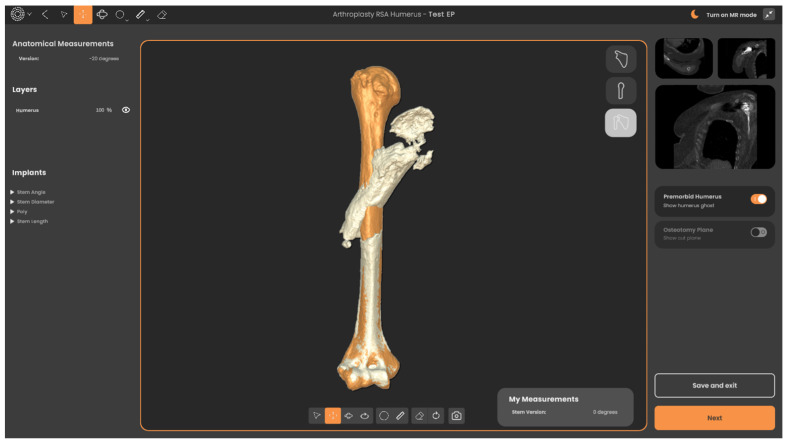
Image from Reflect^TM^ from Akunah (Brisbane, Australia) showing the pathologic humerus overlayed on the mirrored contralateral humerus (orange) with the implant in situ subtracted to allow for an accurate assessment of the bony deficiency.

Traditionally, contralateral radiographs of the whole humerus marked with magnification markers are used to gauge the extent of proximal humeral bone deficiency in revision surgery. However, this method only allows for a two-dimensional assessment of the defect, provided that the contralateral humerus is unaffected by any pathology or has not undergone an arthroplasty. Alternatively, advanced preoperative planning software can be used for this purpose. In our series, the senior author uses the Reflect Complex Revision Planning Service from Akunah (Brisbane, Australia). Metal artifact reduction coupled with automatic landmarking using artificial intelligence (AI) algorithms allows for effective metallic implant subtraction and the segmentation of preoperative computed tomography (CT) scans. Three-dimensional (3D) models of the whole humerus being revised, with and without its pre-existing implants, are created to allow for clear circumferential visualization of bony deficiencies (Figure 1). The contralateral humerus, including both epicondyles, is fully imaged, mirrored, and overlaid onto the affected humerus to highlight the humeral length and height that needs to be restored (Figure 2). In cases where the contralateral humerus is pathological or has undergone an arthroplasty, Statistical Shape Modeling (SSM) techniques can be utilized, which operate on a background of AI algorithms to predict the native pre-morbid humeral length and shape. Once the pre-morbid humeral length and shape are obtained, the degree of bony deficiency anticipated after implant removal is assessed, and the level of humeral osteotomy is planned accordingly.

### 2.2. Assessment of Humeral Bony Deficiency

The varying degrees of bony deficiency in the humerus can be identified based on the preoperative 3D models generated above. Generally, the four types of bony deficiency often encountered are as follows:

Bone deficiency proximal to the subscapularis (<4 cm)

Greater tuberosity and proximal humeral fractures are often encountered with bony deficiencies in the region of the tuberosities. This can be addressed by using a cemented fixation stem and bone grafting the proximal epiphyseal region of the implant. The tuberosities are then mobilized and reattached onto the cemented stem–bone graft complex with morselized bone allografts in between to facilitate the healing of tuberosities onto the implant. An autologous iliac crest graft is a suitable alternative as well. A standard stemmed implant with a length of around 90 mm can usually be used.

Bone deficiency at the level of the pectoralis major, involving the latissimus dorsi (4–7 cm)

**Figure 3:**
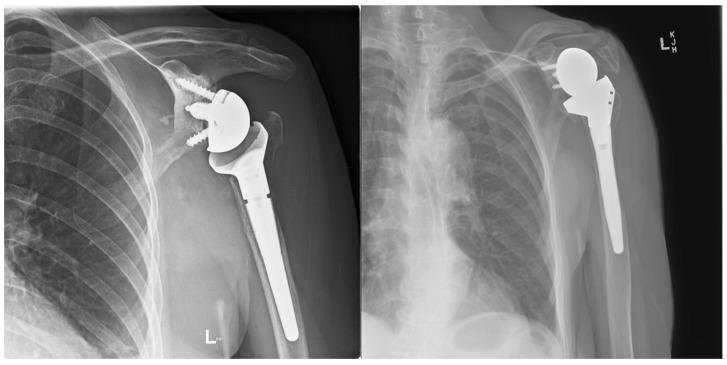
Preoperative X-rays showing proximal stress shielding and bony resorption above the level of the pectoralis major insertion.

This is usually associated with a humeral stem that is well fixed distally, which has led to proximal stress shielding and subsequent bony resorption above the level of the pectoralis major insertion (Figure 3). Commonly, a sliver of bone into which the pectoralis major, teres major, and latissimus dorsi tendons are inserted is intact. The greater and lesser tuberosities are usually reduced to shell-like bony fragments with remnants of the posterior cuff and subscapularis tendons attached. These muscle–tendon complexes with bony attachments are identified, mobilized, and tagged.

**Figure 4:**
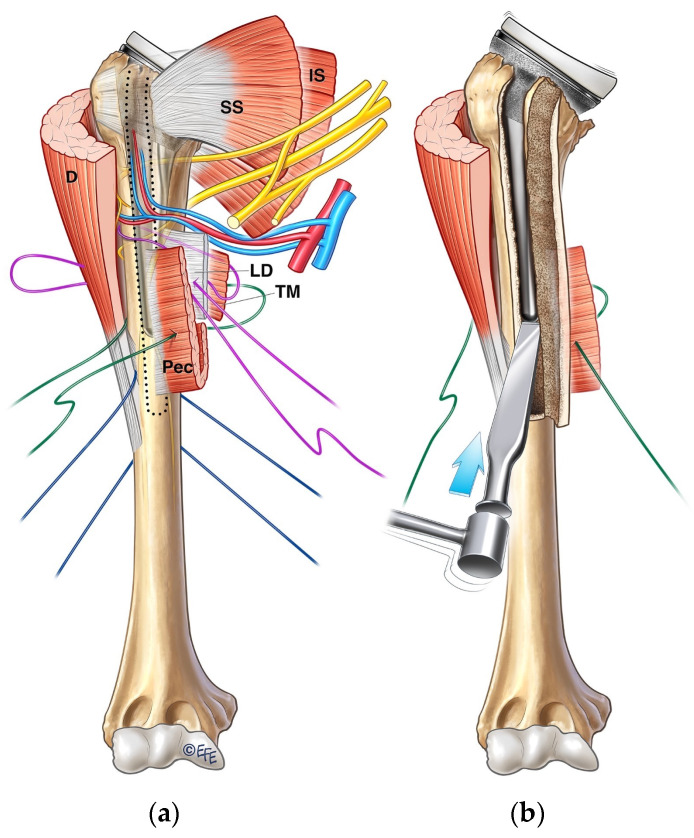
(**a**) Long window humerotomy being carried out from the bicipital groove to the site of pectoralis major tendon insertion (Pec), with cerclage suture placement including Pec, latissimus dorsi (LD), and teres major (TM) tendons. (**b**) Preservation of Pec, LD, and TM tendon attachments en bloc during disimpaction of well-fixed stem. (D, deltoid; SS, supraspinatus; IS, infraspinatus).

The revision of these cases, such as in periprosthetic fractures, requires removing the humeral stem. Depending on the fixation technique and how well fixed it is, often a humerotomy is required to disimpact the stem. The bicipital groove is identified, and a long humerotomy is performed from the tip of the remaining proximal tuberosity until about 2 cm distal to the tip of the stem. The intraosseous part of the stem can be “palpated” using a Kirschner wire drilled sequentially from proximal to distal until it goes past the tip of the stem, and that guides the extent of the humerotomy. Once the long humerotomy is performed, flexible osteotomes are used around the stem to disimpact it proximally (Figure 4). The pectoralis major insertion with its osseous attachment is osteotomized out en bloc, creating a window through which the stem can be easily removed. The region of the humerus where the window humerotomy was carried out is reconstructed by passing a cerclage suture or tape circumferentially around the humerus to include the latissimus dorsi and pectoralis major insertions and their respective bony attachments. Care is taken to dissect and protect the radial nerve during this stage. If the degree of bone loss assessed intraoperatively permits a stable revision construct, one can proceed by cementing the revised humeral stem in a more proximally seated position and combining it with a large, eccentric glenosphere. This construct combination should achieve adequate humeral height and allow for appropriate soft-tissue tensioning of the revision construct. Otherwise, if the degree of bone loss is too extensive, an APC or tumor prosthesis should be considered.

Bone deficiency distal to the pectoralis major tendon insertion (7–13 cm)

A defect where the segmental bone loss is below the pectoralis major insertion cannot usually be addressed by keeping the humeral stem proud. Instead, reconstruction of the proximal humeral deficiency needs to be performed using an APC or a tumor prosthesis [10,11]. In these cases, it is paramount to preserve the deltoid insertion onto the native humerus at all costs, as this significantly affects the postoperative flexion and abduction.

Bone deficiency distal to the deltoid insertion (>13 cm)

The deltoid insertion may be compromised in cases of significant proximal deficiency of more than 13 cm [8,12,13]. If an APC has to extend distal to the deltoid insertion, the patient’s postoperative elevation is usually not as satisfactory as when the native deltoid insertion is still preserved on the humerus. Either way, in the case where a large APC construct is required, it is imperative to reattach the deltoid insertion onto the APC and have it tensioned with the arm at 30° of abduction. Meticulous restoration of the humeral height and soft-tissue reattachment, as detailed below, is imperative for a stable construct.

### 2.3. Humeral Allograft Sizing and Selection of Revision Implants

**Figure 5:**
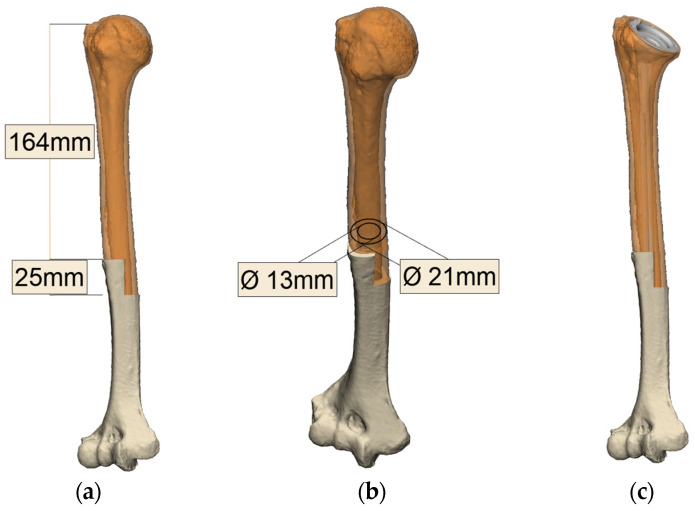
Preoperative plan with the native humerus overlayed on the pre-morbid humerus (orange)**.** (**a**) The length of the bony deficiency is determined by using the predicted pre-morbid humerus as a reference; (**b**) the diaphyseal diameter at the level of the planned osteotomy site is measured preoperatively to ensure the allograft–humerus construct can accommodate the distal portion of the humeral revision stem; (**c**) the position of the chosen implant is planned after determining the appropriate proximal humerus reconstruction and humeral height.

In cases with significant proximal humeral bone loss or when an APC is required, a one-size-fits-all implant generally does not work. Humeral deficiency and its characteristics are evaluated and matched with commercially available implants to achieve the best size fit. When using an APC construct, a size-matched humeral allograft is sourced from the bone bank. A slightly oversized humeral allograft may also be used to allow for overlapping of the humeral allograft step-cut osteotomy onto the native humerus. The diaphyseal diameters of the humeral allograft and native humerus at the level of the planned osteotomy site are measured to ensure the allograft–humerus construct can accommodate the distal portion of the humeral revision stem (Figure 5). Preoperative planning to restore the humerus length and height using an appropriately sized humeral allograft is paramount to ensure the stability of the APC-RSA construct.

## 3. Surgical Technique

### 3.1. Exposure and Explantation

The patient is placed safely in a beach chair position, and routine induction of general anesthesia is carried out. This involves the administration of prophylactic antibiotics and tranexamic acid (TXA), an interscalene catheter for effective pain relief, and arterial pressure and cerebral blood flow monitoring throughout the operative period. All revision cases are carried out via a two-step, single-stage approach, as described by Italia et al. [10]. Step One is exposure, explant, and thorough debridement. This involves an extended deltopectoral approach in which all scar tissue is debrided, and the subscapularis, latissimus dorsi, teres major, and pectoralis major tendons, with their osseous attachments, if present, are identified and preserved. Plexus dissection and axillary nerve neurolysis are carried out aided by the surgical loupes worn and the use of a nerve stimulator. Complete glenoid visualization is achieved via 360° peri-capsular release. The explant of the glenoid and humeral implants is determined based on an intraoperative assessment corresponding with the preoperative plans. In revision cases with a well-fixed glenoid component, properly positioned implants may be retained. Otherwise, a well-fixed but malpositioned glenoid baseplate must be explanted meticulously to preserve as much glenoid bone stock as possible. A glenoid neck window osteotomy, as described by the senior author, may be necessary, wherein a 1 cm × 1 cm bone window is created medially to the tip of the peg along with sequential disimpaction of the undersurface of the baseplate [10]. Attempts to lever the baseplate out must be avoided. This allows for the safe removal of a well-fixed baseplate. After explantation, the degree of bone loss on the glenoid and humeral side is then assessed, and various modalities of reconstruction and fixation are planned accordingly. The joint and wound are then thoroughly washed using pulse lavage. Two grams of vancomycin powder are applied, and the skin is closed with a No. 1 nylon suture. A complete clean changeover then occurs, wherein the surgical team re-scrubs, the patient is re-draped, and a second set of clean instruments is used.

### 3.2. Glenoid Implantation

Step Two of the single-stage approach involves glenoid and humerus reconstruction and implantation. In primary cases, the glenoid is prepared in a standard fashion. In revision cases, re-assessment of the glenoid defect after explantation is conducted. The 3D glenoid bone loss can be quantified using Gupta–Seebauer classification [14]. The restoration of the native glenoid joint line is paramount [15,16]. This can be achieved using bone grafts or metallic augments. The senior author prefers reconstructing significant glenoid defects using bone grafts [17]. The “50% rule” is adhered to when bone grafting and implanting the new glenoid metaglene to achieve a stable glenoid construct [14].

### 3.3. Humeral Allograft Preparation

**Figure 6:**
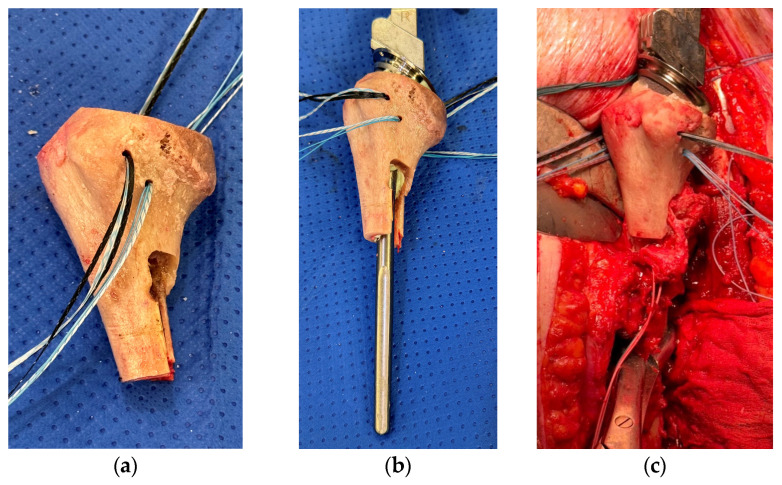
Preparation of the APC at the back table. (**a**) A step-cut osteotomy is performed on the allograft after determining the appropriate length needed for reconstruction. Multiple all-suture anchors to be used for the tendon reattachments are inserted into the proximal humeral allograft prior to cementing and inserting the definitive humeral prosthesis. (**b**) The chosen humeral stem is cemented into the proximal humeral allograft to form the APC construct. (**c**) Cementing of the APC construct onto the native humerus.

A size-matched humeral allograft is usually prepared on the back table. Standard humeral preparation of head resection and diaphyseal reaming is carried out based on the choice of implant. The level and length of the humeral allograft osteotomy are checked against preoperative plans. However, it can be adjusted intraoperatively to allow for unexpected bone loss on the native humerus. Restoring the humeral length and height to pre-morbid status at this step is paramount. A step-cut osteotomy is then performed on the humeral allograft (Figure 6a). This is the senior author’s preferred method of securing the APC construct to the native humerus compared to the more widely used de-rotation plating technique. This allows for a more stable fixation, especially in rotation, and provides a larger surface area for bony contact. As a guide, we recommend the vertical limb for the step-cut osteotomy to be a minimum of 4 cm. A matching step-cut osteotomy is then marked and executed on the native humerus.

### 3.4. APC Trial and Finalization of Humeral Length

The humeral implant trial component with the smallest-sized stem and epiphysis is then inserted into the humeral allograft. Ideally, the length of the stem used should allow for at least 4 cm of fixation in the native humerus. The APC–humerus trial construct is then positioned on the native humerus and held on with a Verbrugge bone clamp at the level of the step-cut osteotomy. The construct is then reduced to assess joint stability and confirm the restoration of the humeral length and height. Depending on the chronicity of the injury, the exact length of the humeral allograft may need to be adjusted at this stage. In an acute situation, a size-to-size matched allograft is often attained based on preoperative planning, as described above. However, in cases with a chronic history of bone loss, the soft-tissue contractures do not allow for a size-matched humeral allograft. In the senior author’s experience, shortening the APC complex by 15–20% of its pre-planned normal length is often necessary. This takes into account that RSA generally distalizes the joint by approximately 3 cm. After intraoperative trialing and assessing the feedback from the tension at the joint, the final length of the humeral allograft is then determined and fine-tuned accordingly at the host–graft junction with a micro sagittal saw.

### 3.5. Humeral Allograft Preparation for Soft-Tissue Reattachment

Once the allograft length and height are confirmed, it is deconstructed at the back table with the humeral trial component removed. Multiple all-suture anchors (2.9 mm Juggerknot^TM^ Zimmer Biomet, Warsaw, Indiana) are inserted into the proximal humeral allograft to provide anchor points for the reattachment of soft tissues when the allograft used is devoid of any tendon stumps (Figure 6a,b). Rotator cuff tendons can be reattached onto the tuberosities, and the latissimus dorsi, teres major, and pectoralis major tendons, which were detached earlier with their bony insertions, can be wrapped around the humeral allograft using these sutures to allow for firm union of the tendon–bone unit onto the APC construct. The deficiency of a posterior cuff necessitates a latissimus dorsi tendon transfer for stability and function.

### 3.6. APC Assembly and Fixation

**Figure 7:**
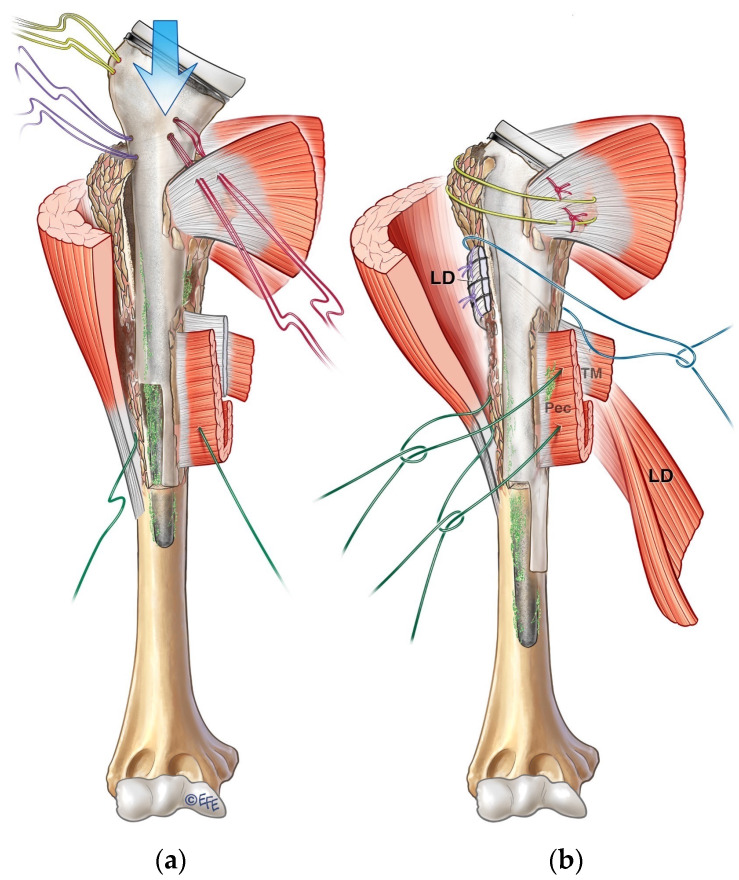
(**a**) Cementing of APC construct onto native humerus. (**b**) Reattachment of tendons: posterosuperior cuff and subscapularis, latissimus dorsi (LD), pectoralis major (Pec), and teres major (TM).

The chosen humeral stem is cemented into the proximal humeral allograft to form the APC construct (Figure 6b). The APC construct is then fixed onto the native humerus with a second mix of cement (Figure 6c and Figure 7). The graft–host junction is bone grafted before the APC is completely impacted into the humerus to prevent the extravasation of cement and allow a bone-to-bone bridge to form between the APC and the native humerus. Because of the step-cut osteotomy, the fixation is usually stable enough; hence, it does not typically require any additional fixation, provided an adequate stem length of at least 4 cm is also achieved distally in the native humerus. The joint is reduced with a trial liner to check for appropriate tension and stability. It is always important to check the tension of the brachial plexus at this stage to ensure that the length of the construct does not cause excessive tension on the nerves to avoid plexopathy postoperatively. The appropriate thickness of polyethylene liner is then used, and the final construct is reduced.

### 3.7. Tendon Reattachment and Tendon Transfers

Meticulous reattachment of the latissimus dorsi, teres major, and pectoralis major tendons is carried out using circumferential suture loops around the APC construct (Figure 7b). The subscapularis is reattached with the arm held at 30° external rotation, while the posterosuperior cuff is reattached with the arm internally rotated to 45°. Reattachment of the posterosuperior cuff is critical in both primary RSA and revision cases, as failure to do so can lead to postoperative instability. If the posterosuperior cuff is deficient, then a latissimus dorsi transfer is carried out from the anterior to the posterolateral aspect of the APC construct. A lower trapezius tendon transfer is the next option to provide posterior restraint for the revised arthroplasty if the latissimus dorsi cannot be transferred. Once all soft-tissue reattachment is completed, the joint is thoroughly washed with dilute betadine solution, 2 g of vancomycin powder is applied, and the wound is meticulously closed after hemostasis.

### 3.8. Postoperative Protocol

The shoulder is immobilized using a brace with 60° abduction (Omo Immobil, Ottobock, Duderstadt, Germany) for 6 weeks. Usually, compression bandaging for edema management is instituted from day one postoperatively up to two weeks due to significant swelling in the arm after such an extensive revision procedure. Diligent care of the wound is needed to ensure that the wound is completely dry. If the wound has increased seropurulent drainage postoperatively, a simple negative pressure wound therapy dressing system such as a PICO (Smith+Nephew, Watford, UK) dressing is applied. Postoperative rehabilitation begins on postoperative day one with active range of motion of the elbow, hand, and wrist. At the two- to three-week mark, the patient is reviewed in the clinic, and wall walks are commenced within the patient’s comfort zone while continuing with full active range of motion of the elbow, hand, and wrist. The patient is allowed to use their hand for toileting, showering, computer work, and eating out of the brace. The postoperative rehabilitation must be individualized based on the patient’s co-morbidities, age, cognitive capacity, and the extent of surgery. The senior author prefers to commence active rehabilitation and early mobilization as soon as possible. Of note, it is crucial to maintain the patient in a 60°-abduction brace for six weeks to allow the latissimus dorsi, teres major, and pectoralis major muscles to stay outstretched and avoid any abnormal scapulothoracic kinematics when rehabilitation progresses.

## 4. Outcomes After Revision RSA with APC

Patients who underwent revision RSA with an APC for proximal humeral bone deficiency were identified from a single-surgeon, single-institution prospectively collected database from August 2016 to February 2023. Five patients (mean age: 73; range: 61–82) were available for follow-up. As part of standard clinical practice, patient-reported and clinical outcomes were collected preoperatively, at six months, one year, and two years postoperatively using a data collection platform (Akunah PROMs, Akunah, Brisbane, Australia). Patient-reported outcome measures (PROMs) included the pain score using the visual analogue score (VAS), functional outcome using the American Shoulder and Elbow Surgeons (ASES) shoulder score, and satisfaction before and after the surgery. Active range of motion (ROM) included forward flexion, lateral elevation, and external rotation in adduction. CT scans were performed at ten weeks postoperatively to assess graft healing and union and at two years postoperatively to assess for osteolysis and loosening.

### 4.1. Preliminary Results

All patients were female, and all cases were revisions referred to the senior author. The reasons for revision are presented in Table 1. Three of the five cases had already undergone a revision before being referred to us. Three of the cases had over five previous failed RSA attempts due to instability.

Four cases were performed as a single-stage procedure and one case as a two-stage procedure because of previous history of multi-bacterial colonization following a primary RSA. Four cases had 5 cm of proximal humeral bone loss, while one with a periprosthetic humeral fracture had 20 cm of bone loss.

After a mean follow-up of 6 months (range: 3–24), the pain score decreased from 5 to 3. Forward flexion improved from a mean of 53° to 70° (range: 40–90°). External rotation in adduction also improved from 0° to 22° (range: 20–25°) (Table 2). One patient was lost to follow-up after their initial 6-week postoperative review due to the medical morbidity of a stroke.

### 4.2. Radiologic Follow-Up

**Figure 8:**
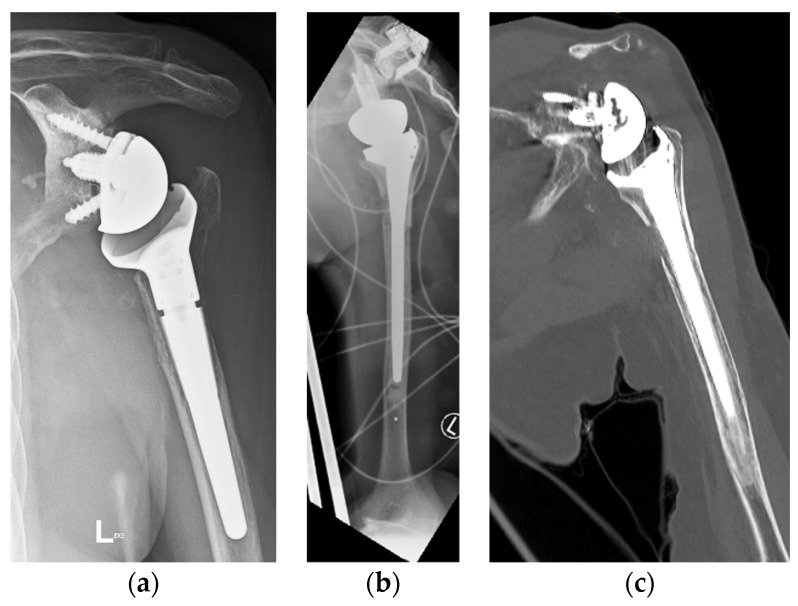
(**a**) Preoperative anteroposterior (AP) X-ray showing bone loss on the proximal humerus; (**b**) immediate postoperative AP X-ray showing reconstruction of the proximal humeral defect using an APC; (**c**) coronal CT scan showing union of the APC with the native humeral shaft.

Two patients had repeat CT scans at two years, and one had X-ray at four years, showing complete graft union and no osteolysis (Figure 8). As mentioned, one patient was lost to follow-up, and another refused follow-up imaging.

### 4.3. Complications

One of the patients had a periprosthetic fracture below the tip of the stem after a fall one year postoperatively, which was successfully managed with fixation augmented with a strut allograft. A CT scan at 12 months post-fixation of the APC periprosthetic fracture showed union at the fracture site. No cases were revised for graft nonunion, graft resorption, or infection.

## 5. Systematic Review

### 5.1. Methods

**Figure 9:**
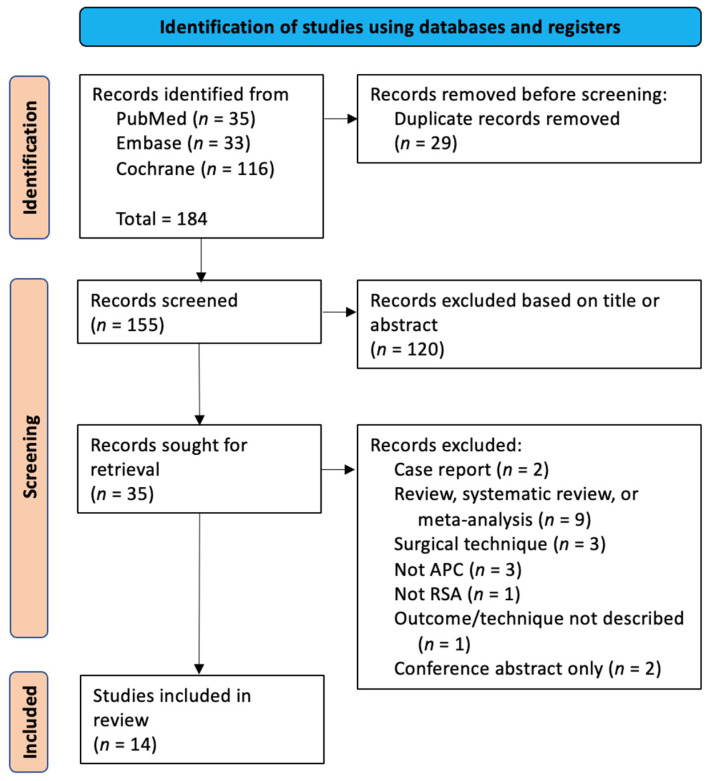
Preferred Reporting Items for Systematic Reviews and Meta-Analyses flow diagram depicting article identification, subsequent exclusions, and analysis for techniques and complications. APC = allograft prosthetic composite; RSA = reverse shoulder arthroplasty.

A systematic review was conducted following the Preferred Reporting Items for Systematic Reviews and Meta-Analyses (PRISMA) guidelines [18]. A comprehensive search of the PubMed, Embase, and Cochrane databases was performed by two reviewers (KI and CC), with queries referred to the senior author (AG) from inception to August 1, 2024. Any conflicts were resolved via discussion or referral to the senior author (AG). The following search terms were used: (“proximal” AND “humer*” AND “reverse shoulder”) AND (“allograft” AND “prosthe*” AND “composite”) (see Supplementary Materials for the detailed search strategy). The initial query yielded 184 reports. After removing the duplicates, 155 unique articles remained. The titles and abstracts were manually reviewed to identify papers reporting on the technical details of the use of an APC in revision RSA, with non-relevant articles excluded, resulting in 35 potential articles. Studies categorized as case reports, reviews, systematic reviews or meta-analyses, technical notes, articles without the use of RSA, and conference abstracts were excluded. Ultimately, 14 studies reporting on 255 APC-RSA procedures were included in the systematic review, with the basic patient demographics synthesized and presented and missing data reported as not available. As this review focused on surgical techniques, no risk-of-bias assessment was performed. No additional studies were identified as relevant through the manual search. Studies involving primary APC-RSAs were also included, in addition to APC-RSAs in revision cases, to allow for a comprehensive review of the technical details of all APC-RSAs. A flow diagram outlining the study selection process is provided in Figure 9.

### 5.2. Results

We focused on a comparison of the technical details of the included papers. A total of 14 studies with 255 patients undergoing proximal humerus reconstruction with APC-RSAs were included in our analysis. The mean follow-up of the reporting studies was 57 months, ranging from 24 to 96 months (Table 3). The mean age at surgery was 55 years, ranging from 10 years to 87 years. An oncological pathology, traumatic bone loss, or failed primary reconstructive procedures were indications for APC-RSAs in all included studies (Table 4).

#### 5.2.1. Cause of Bone Loss

The primary causes of bone loss varied across the cohort (Table 4). Seventy-eight (78) patients (31%) had oncological indications for APC-RSA. Additionally, 111 patients (44%) had bone loss due to failed hemiarthroplasties (HA). Thirty-five (35) patients (14%) had failed reverse shoulder arthroplasties (RSA). Eight (8) patients (3%) had experienced failed total shoulder arthroplasties (TSA), while two patients (1%) had undergone failed open reduction and internal fixation (ORIF) procedures, contributing to bone loss.

#### 5.2.2. Type of Allograft

Fresh-frozen humeral allografts were the predominant graft material used in APC procedures. Two hundred thirty-nine (239) out of two hundred ninety-one (82%) APC procedures utilized fresh-frozen (FF) humeral allografts across the studies. Boileau et al. [8] and Cox et al. [22] also used proximal femoral allografts in 11 cases. Additionally, some authors described utilizing surgical mesh [28,29].

#### 5.2.3. Surgical Technique

Most of the APC-RSAs were cemented into the native bone (Table 5). Step-cut osteotomies were performed in 117 patients across the studies. Fixation plates were used in 44 procedures. Other techniques included press-fit fixation with interlocking screws [8], the use of surgical mesh combined with tension band wiring [28], and cables or cerclages (n = 110) [2,5,21,22]. In 14 cases, the L’episcopo procedure was also performed [8,20].

#### 5.2.4. Clinical Outcomes

Different outcome scores were reported in the studies with data synthesis where available using weighted means. Six studies reported VASs, which showed postoperative pain ranging from 1.8 to 3.8 (Table 6). Three studies reported a Constant score, with a mean of 45.7 and a mean adjusted Constant score of 56. Eight studies reported ASES scores, with a mean of 62.9. An SSV was reported in two studies, with a mean of 51. SST was reported in seven studies, with a mean of 4.5. SANE was reported by Cox et al. [22], with a mean of 56.4. ADLER was reported by Callamand et al. [20], with a mean of 21. An MSTS score was reported in five studies, with a mean of 57.6. The mean QuickDASH was 26.4, as reported in two studies. Lazerges et al. [26] reported SF-12, with a PCS of 44.4 and MCS of 39.7, while Antal et al. [19] used SF-36, showing a mean of 67%. A satisfaction rate was reported in six studies, showing a mean satisfaction rate of 87%.

The mean active range of motion was reported in 10 studies, with a mean active forward flexion of 89, external rotation of 21, and abduction of 78. The rest of the clinical outcomes are reported in Table 6.

#### 5.2.5. Radiologic Outcomes

A majority of the studies reported on graft incorporation, with a mean rate of 84% (Table 7). Three studies reported the time to union, with a mean of 6.7 months (range: 6–8 months). Seven studies assessed graft resorption, with a mean rate of 24.5% (range: 0–64%). Scapular notching was reported in seven studies, with a mean rate of 16.9%. The remaining radiological assessments are summarized in Table 7.

#### 5.2.6. Complications

Complications were commonly reported across the studies (Table 7). Dislocation was the most common complication, occurring in 26 cases, followed by periprosthetic or allograft fractures, which was seen in 18 cases. Furthermore, prosthetic loosening occurred in eight cases. Infections were recorded in seven patients. Other complications are reported in Table 7.

#### 5.2.7. Survivorship

Thirteen cases were reported to have undergone revision (Table 7). The mean overall revision-free survival was 90% at 5 years as reported in three studies, and 69% at 10 years as reported in two studies.

## 6. Discussion

The use of an APC to address proximal humeral bone deficiency in revision shoulder arthroplasty has shown acceptable clinical and radiological outcomes, which were achieved by meticulous preoperative planning, the restoration of humeral length, and soft-tissue reconstruction.

Proximal humeral bone deficiency in revision shoulder arthroplasty is a complex problem and presents a significant challenge to surgeons. Significant bone loss at the proximal humerus predisposes the revised construct to a cascade of failure. Deficient sites for reattachment of the rotator cuff and proximal humerus musculature lead to poor soft-tissue tensioning, contributing to instability and dislocation [30,31]. The remaining humeral length is often shorter than ideal, causing the revised humeral stem to be uncovered proximally with only distal fixation. This subjects the stem to high torsional and bending forces of the humerus, leading to early loosening of both the humerus and glenoid components [32]. It is anticipated that this problem will be more emergent given the increasing use of RSA worldwide [33,34]. Gregori et al. carried out a systematic review on the use of APC in proximal humeral bone loss, which included ten studies and a total of 239 patients showing favorable clinical outcomes and APCs as a valid option in dealing with this challenging problem [35]. Our systematic review provides an updated assessment of the literature, including 14 studies and 255 patients, offering a more recent evaluation of APCs in RSA, with a greater focus on technical details and complications.

Options for managing proximal humeral bone loss during revision arthroplasty include the use of allografts or proximal humerus-replacing implants such as tumor endoprosthesis [36]. Proximal humerus-replacing implants are commonly used following tumor resections but have also been used as a salvage procedure during revision shoulder arthroplasty when proximal humeral bone loss is present [37]. Tumor endoprosthesis mitigates the risk of nonunion, osteolysis, or graft failure associated with the use of allografts. However, the advantage of using allografts over endoprosthesis is the biologic reconstruction of the proximal humerus, allowing for bone stock restoration, as well as biologic healing of reattached soft tissues or tendon transfers. Our series has shown good graft incorporation, with no cases being revised for graft failure. The systematic review also revealed high graft incorporation in the literature, with a mean rate of 84%, as well as a high mean overall revision-free survival rate of 90% at 5 years, which decreases to 69% at 10 years.

To meticulously address the challenges associated with reconstruction related to proximal humeral bone deficiency, this technical note has highlighted the importance of preoperative planning to accurately determine the amount of bone that needs to be reconstructed to restore the humeral height. The contralateral humerus may be used as a reference, as recommended by Boileau et al. [8] and Sanchez-Sotelo et al. [9] in their techniques. However, if this is not available, the use of preoperative planning software that has the ability to perform metal artifact reduction and provide humeral SSM is proposed. Aside from giving insights into the extent of bone loss, it allows the revision surgery to be performed in a single-stage procedure, which has been shown to be clinically and economically advantageous [10]. Our systematic review reveals that preoperative planning for humeral length and height is not consistently performed across the included studies, which may have contributed to the risk of postoperative instability, which was the most common complication seen in our systematic review. Complications related to inadequate tension, such as instability, are frequently associated with failure to restore humeral height, and/or improper implant positioning and alignment, all of which can be prevented by meticulous preoperative planning.

The importance of appropriate tensioning of the soft tissues by reattachment onto the humerus, which provides stability to the revised RSA-APC joint complex, has also been highlighted. This key point is echoed by other published case series in the literature [5,8,9]. An RSA has been informally spoken of as a “soft-tissue” operation. There is an equal, if not greater, focus on getting the soft-tissue tensioning right besides bony cuts and the optimum placement of prostheses. In primary cases, soft-tissue focus is on capsular release at the posterior–inferior part of the humerus anatomical neck and 360° peri-glenoid. This includes the long head of triceps insertion at the infraglenoid tubercle. The subscapularis is detached from superior glenohumeral ligament attachments to allow for smooth subcoracoid gliding movement. Tension on the axillary nerve and plexus can be used as a guide intraoperatively whether the construct has been overlateralized or distalized. For closure, the subscapularis is always repaired in a double-row fashion, and the posterior cuff is always reattached. If not possible, a latissimus dorsi tendon transfer is carried out. Similarly, all the above steps apply in revision cases using an APC. In addition, the focus is on debulking scar tissue, nerve neurolysis, and protection of the axillary and radial nerves. Soft-tissue balance now focuses on restoring posterior constraint, beginning with posterior cuff reattachment, latissimus dorsi tendon transfer, or lower trapezius transfer if necessary. This meticulous focus on soft-tissue reattachments and reconstruction in both primary and revision shoulder arthroplasties allows for the achievement of a stable construct and leads to a better range of motion and clinical outcomes.

This technical note demonstrated an all-suture anchor method of reattaching the remaining rotator cuff tendons and tendon transfers onto the proximal humeral allograft, negating the need for the transosseous drilling of bone tunnels. This technique may be helpful in countries where only denuded bony allografts can be sourced, compared to countries like the USA, where bony allografts with attached tendon complexes are readily available. We also highlight the importance of preserving the patient’s native tendon–bone complexes and their subsequent reattachment onto the humeral allograft via circumferential suture loops to allow for osseous integration.

In our series, most cases involved the revision of revision RSA. Two patients had undergone over six previous operations. These are a difficult cohort of patients with unsatisfactory preoperative function, who are less likely to do as well as those who had primary shoulder arthroplasties. These patients showed improvements in their clinical scores and range of motion, although the gains were modest. Our findings align with those presented by Gregori et al. [35], who carried out a systematic review of the use of APCs in proximal humeral bone loss on 239 patients. Favorable clinical outcomes were observed, although the range of motion and patient and PROMs were also not as excellent as what is expected after a primary RSA, with a mean forward flexion of 90°, abduction of 71°, external rotation of 21°, and internal rotation of 4°. These results are also consistent with our systematic review, which showed that shoulder reconstruction with an APC provides acceptable shoulder function and a high satisfaction rate despite only modest improvements in the range of motion and PROMs. The limitations of the systematic review include examining only the technical aspects of the procedure in a specific group of patients, which limited the scope of the review. However, this was due to the relevance to the specific details of the case series presented.

The shortcomings of the case series were the retrospective nature of this case series and the small sample size. The clinical outcomes presented were also short term, with a mean follow-up of 6 months. A larger series with longer follow-up is necessary to validate the results. Despite these limitations, this technical note discussed, in detail, the preoperative and intraoperative techniques and tips necessary to perform proximal humeral reconstruction using APC, which would be clinically relevant in cases of severe proximal humeral bone deficiency encountered during revision RSA.

## 7. Conclusions

The use of an APC construct is a viable option for proximal humeral bone deficiency in revision shoulder arthroplasty. The restoration of the pre-morbid humeral length and height, with appropriate tensioning via conscientious soft-tissue reattachment, is critical to maintaining stability in the revised RSA-APC joint complex and achieving acceptable outcomes.

## Figures and Tables

**Table 1:** Demographics of patients who underwent RSA with APC for proximal humeral bone deficiency.

Variable	*n* (%)
Sex	
Female Male	5 (100%) 0 (0%)
Surgery	
Primary Revision One-stage Two-stage	0 (0%) 5 (100%) 4 (80%) 1 (20%)
Reason for revision Infection Pain Instability Periprosthetic fracture	1 (20%) 1 (20%) 1 (20%) 2 (40%)
Proximal humeral bone deficiency (cm)	8 (range 5–20)

**Table 2:** Clinical and functional outcomes.

Parameters	Preoperative	Postoperative
VAS (mean ± SD)	5 ± 3	3 ± 8
Range of motion (mean ± SD)		
Forward flexion (deg) Lateral elevation (deg) External rotation (deg)	53 ± 40 33 ± 40 0 ± 0	70 ± 26 80 22 ± 3
PROMs (mean ± SD) ASES	32 ± 18	35 ± 21
Satisfaction (%)	0%	50%

**Table 3:** Study details.

Articles	Study Type	Mean Follow-Up (mo)	No. of Patients	Mean Age (yr)
Antal et al., 2023 [19]	Retrospective	96	12	48.5
Boileau et al., 2020 [8]	Retrospective	48	25	59
Callamand et al., 2020 [20]	Retrospective	30	11	51
Chacon et al., 2009 [5]	Prospective	30	25	nA
Cox et al., 2019 [21]	Retrospective	68	73	67
Cox et al., 2024 [22]	Retrospective	53	14	67
Han et al., 2022 [23]	Retrospective	44	6	47
Houdek et al., 2021 [24]	Retrospective	84	10	54
Houdek et al., 2022 [25]	Retrospective	72	11	51
Lazerges et al., 2017 [26]	Retrospective	71	6	66
Levy et al., 2007 [2]	Retrospective	24	8	nA
Sanchez-Sotelo et al., 2017 [27]	Retrospective	64	26	62
Zhou et al., 2024 [28]	Retrospective	81	8	37
Zuo et al., 2021 [29]	Retrospective	34	20	46

RSA, reverse shoulder arthroplasty; mo, months; yr, years; nA, not available.

**Table 4:** Technical details of studies.

Articles	Cause of Bone Loss (n)	Type of Allograft (n)
Antal et al., 2023 [19]	Oncological, primary (12)	nA
Boileau et al., 2020 [8]	Oncological, primary (2); failed megatumorprosthesis (6); failed RSA (12); failed HA (5)	FF humeral allograft (24); femoral allograft (1)
Callamand et al., 2020 [20]	Oncological, primary (11)	FF humeral allograft
Chacon et al., 2009 [5]	Failed HA (24); failed bipolar HA (1)	FF humeral allograft
Cox et al., 2019 [21]	Failed HA (54); failed RSA (17); failed TSA (1); failed ORIF (1)	FF humeral allograft, remnant subscapularis tendon
Cox et al., 2024 [22]	Failed HA (3); failed TSA (3); failed RSA (7); failed ORIF (1)	FF humeral allograft (4); FF proximal femur allograft (10)
Han et al., 2022 [23]	Oncological, primary (6)	FF humeral allograft
Houdek et al., 2021 [24]	Oncological, primary (10)	FF humeral allograft with tendons
Houdek et al., 2022 [25]	Failed APC-HA (4); failed HA endoprosthesis (5); failed osteoarticular allograft (2)	FF humeral allograft with tendons
Lazerges et al., 2017 [26]	Oncological, primary (6)	FF humeral allograft
Levy et al., 2007 [2]	Failed HA (8)	FF humeral allograft, remnant subscapularis tendon
Sanchez-Sotelo et al., 2017 [27]	Oncological, primary (3); fracture (5); failed HA (11); failed TSA (4); failed RSA (3)	FF humeral allograft with tendons
Zhou et al., 2024 [28]	Oncological, primary (8)	FF humeral allograft with surgical mesh
Zuo et al., 2021 [29]	Oncological, primary (20)	Devitalized autograft

RSA, reverse shoulder arthroplasty; HA, hemiarthroplasty; TSA, anatomic total shoulder arthroplasty; ORIF, open reduction and internal fixation; nA, not available; FF, fresh frozen.

**Table 5:** Surgical technique.

Articles	Surgical Technique (n)
Antal et al., 2023 [19]	nA
Boileau et al., 2020 [8]	Cemented (15); press fit (2); press fit with interlocking screws or bolts (8); step-cut osteotomy (nA); L’episcopo procedure (9)
Callamand et al., 2020 [20]	Cemented (11); fixation plate (4); Chevron osteotomy (7); L’episcopo procedure (5)
Chacon et al., 2009 [5]	Cemented (25); step-cut osteotomy (25); two 1.7 mm cables (25); fixation plate (1)
Cox et al., 2019 [21]	Cemented (73); step-cut osteotomy (73); multiple 1.7 mm cables (73); remnant subscapularis tendon attached (73)
Cox et al., 2024 [22]	Cemented (14); step-cut osteotomy (10); transverse osteotomy (3); transverse osteotomy + telescoping of graft (1); cerclage cables (4), fixation plate-and-screw construct (3)
Han et al., 2022 [23]	Cemented (6); telescoping technique (2)
Houdek et al., 2021 [24]	nA
Houdek et al., 2022 [25]	Cemented (11); fixation plate (10); step-cut osteotomy (1)
Lazerges et al., 2017 [26]	Cemented (6)
Levy et al., 2007 [2]	Cemented (8); cerclage cables (8); step-cut (8); remnant subscapularis tendon attached (8)
Sanchez-Sotelo et al., 2017 [27]	Cemented (26); fixation plate (26) remnant rotator cuff attached if possible
Zhou et al., 2024 [28]	Cemented (8); surgical mesh (nA); tension band wiring (1)
Zuo et al., 2021 [29]	Cemented (20); autograft tendon reattachment (nA); surgical mesh (nA)

nA, not available.

**Table 6:** Clinical outcomes.

Articles	Pain	ASES Score	SST	MSTS Score	Others	ROM	Satisfaction
Antal et al., 2023 [19]				84%	**SF-36:** 67%	Flexion: 120–170 Abduction elevation: 140–160 Extension: 20–30 IR/ER: 10–30	
Boileau et al., 2020 [8]	**VAS:** Preoperative: 6 Postoperative: 2				**Constant score:** Preoperative: 21; postoperative: 42 **Adjusted Constant score:** Preoperative: 24; postoperative: 53 **SSV** Preoperative: 20; postoperative: 50	Preoperative: Forward elevation: 50 ER: 0 IR, points: 2 Postoperative: Forward elevation: 90 ER: 10 IR, points: 4	
Callamand et al., 2020 [20]					**Constant score:** 49 **Adjusted Constant score:** 59 **SSV:** 52 **ADLER:** 21	Anterior elevation: 105 ER1: 23 ER2: 21 IR1, points: 4	Very satisfied: n = 9 Satisfied: n = 4
Chacon et al., 2009 [5]		Preoperative: 31.7 Postoperative: 69.4	Preoperative: 1.4 Postoperative: 4.5			Preoperative: Forward flexion: 32.7 Abduction: 40.4 ER: 9.9 IR: sacrum Postoperative: Forward flexion: 82.4 Abduction: 81.4 ER: 17.6 IR: L4	Good or excellent: 76% Satisfactory: 20% Unsatisfied: 4%
Cox et al., 2019 [21]		Preoperative: 33.7 Postoperative: 51.1	Preoperative: 1.3 Postoperative: 3.5			Preoperative: Forward flexion: 49 Abduction: 45 ER: 15 IR, points: 2 Postoperative: Forward flexion: 75 Abduction: 72 ER: 16 IR, points: 3	Good to excellent: 70% Satisfactory: 17% Unsatisfied: 13%
Cox et al., 2024 [22]	**VAS:** 3.8 ± 3.4	56.5 ± 24.9			**SANE:** 56.4 ± 20.8		
Han et al., 2022 [23]	**VAS:** 2.3 ± 1.0		6.2 ± 1.7	25% ± 3.9		Forward flexion: 102.5 ± 10.4 ER: 23.3 ± 19.7 IR: 9.5 ± 1.9	
Houdek et al., 2021 [24]		72 ± 10	6 ± 2	80% ± 9		Forward elevation: 100 ± 39 ER: 34 ± 11	Satisfied: 100%
Houdek et al., 2022 [25]	**Pain:** Preoperative: - Moderate/severe: 100% - None/mild: 0% Postoperative: - Moderate/severe: 18% - None/mild: 82%	Preoperative: 39 ± 13 Postoperative: 58 ± 14	Preoperative: 3 ± 2 Postoperative: 4 ± 2	Preoperative: 41% ± 10 Postoperative: 69% ± 14		Preoperative: Forward elevation: 39 ± 12 ER: 13 ± 11 Postoperative: Forward elevation: 65 ± 41 ER: 25 ± 15	
Lazerges et al., 2017 [26]	**VAS:** At rest: 1.2 During activity: 2.3			73%	**QuickDASH:** 41.2 **Constant score:** 46.1 **SF-12** Preoperative: PCS: 44.4; MCS: 39.7 Postoperative: PCS: 45.5; MCS: 48.4	Active ROM: Forward flexion: 95 Abduction: 57 ER: 8 IR: L4 Passive ROM: Forward flexion: 113 Abduction: 75 ER: 31 IR: L4	
Levy et al., 2007 [2] * results are combination of those treated with RSA-APC and RSA alone		Preoperative: 22.3 Postoperative: 52.1	Preoperative: 0.9 Postoperative: 2.6			Preoperative: Forward flexion: 38.1 Abduction: 34.1 ER: 11.2 Postoperative: Forward flexion: 72.7 Abduction: 70.4 ER: 17.6	Good or excellent: 75% Dissatisfied: 25%
Sanchez-Sotelo et al., 2017 [27]	**Pain:** Overall: Preoperative: - Moderate/severe: 80.8% - None/mild: 19.2% Postoperative: - Moderate/severe: 3.8% - None/mild: 96.2% Primary APC: Preoperative: - Moderate/severe: 100% - None/mild: 0% Postoperative: - Moderate/severe: 0% - None/mild: 100% Revision APC: Preoperative: - Moderate/severe: 72.2% - None/mild: 27.8% Postoperative: - Moderate/severe: 5.6% - None/mild: 94.4%	66.1	4.4		**Strength:** Overall: Preoperative: 3.2 ± 0.2 Postoperative: 3.8 ± 0.1 Primary APC: Preoperative: 3.3 ± 0.2 Postoperative: 3.9 ± 0.3 Revision APC: Preoperative: 3.2 ± 0.2 Postoperative: 3.9 ± 0.2	Overall: Preoperative: - Elevation: 41 ± 8 - ER: 11 ± 3 Postoperative: - Elevation: 98 ± 9 - ER: 31 ± 4 Primary APC: Preoperative: - Elevation: 49 ± 18 - ER: 4 ± 6 Postoperative: - Elevation: 114 ± 16 - ER: 24 ± 7 Revision APC: Preoperative: - Elevation: 37 ± 8 - ER: 15 ± 3 Postoperative: - Elevation: 92 ± 10 - ER: 35 ± 5	Excellent: 7 Satisfactory: 10 Unsatisfactory: 9 Improved from preoperative status: Yes: 25 (96%) No: 1 patient Much better: 17 Better: 8 Worse: 1
Zhou et al., 2024 [28]				26% + 1.7	**QuickDASH:** 11.6 + 4.5		
Zuo et al., 2021 [29]	**VAS:** 1.5 + 0.8	78 + 3.5 (12 months)				Forward flexion: 95 ± 5.6 Abduction: 110 ± 10 (at the end of follow-up); 100 ± 7.6 (12 months) ER: 25 ± 4.5	

VAS, visual analog scale; ASES, American Shoulder and Elbow Surgeons; SST, Simple Shoulder Test; MSTS, Musculoskeletal Tumor Society; SF-36, 36-item Short-Form Survey; SSV, Subjective Shoulder Value; ADLER, Activities of Daily Living requiring External Rotation; SANE, Single Assessment Numeric Evaluation; QuickDASH, Quick Disabilities of Arm, Shoulder, and Hand; SF-12, 12-item Short Form Survey; PCS, Physical Component Score; MCS, Mental Component Score; ROM, range of motion; ER, external rotation; IR, internal rotation. * Results are combination of those treated with RSA-APC and RSA alone.

**Table 7:** Radiologic outcomes, complications, and survivorship.

Articles	Complications	Radiologic Outcomes	Survivorship
Antal et al., 2023 [19]	Dislocation/proximal migration (3); infection (0); fracture/nonunion (0)	Nonunion (0)	
Boileau et al., 2020 [8]	Instability (6); glenoid loosening (4); infection (2); humeral host fracture (1); temporary radial palsy (1)	Incorporation: 96%; scapular notching: 48%; humeral stem loosening: 0%	
Callamand et al., 2020 [20]	Dislocation (1)	Humeral allograft consolidation: 73% at mean of 6 months Osteolysis in epi-metaphyseal zone of allograft (Levigne zones 1 and 7): 64%, with onset of absorption at mean of 10 months Bone insertion of L’Episcopo muscle transfer (Levigne zone 2): absorption in 80%, with onset at mean of 8 months Humeral loosening (0) Systematic graft absorption on glenoid side: partial 3, complete 5, without loosening of baseplate Sirveaux grade 1 notching (2)	
Chacon et al., 2009 [5]	Dislocation (1); dislocation with fracture of both allograft and polyethylene component (1); allograft fracture (1); nondisplaced acromion fracture (1)	Inferior scapular notching (0); loosening of glenoid baseplate (0); subluxation (1) Incorporation: 88% in metaphyseal region; 79% in diaphyseal region Non-incorporation without resorption/fragmentation (4)	Revision (1)
Cox et al., 2019 [21]	Periprosthetic fractures (8); dislocation (4); humeral loosening (3); glenosphere dissociations (2); infection (2)	Average bone loss on immediate postop radiograph: 55 mm Incorporation at metaphysis: 53% Incorporation at diaphysis: 84% Humeral stem loosening (10)	Overall reoperation-free survival rate: 88% at 5 years; 78% at 10 years; 67% beyond 10 years Humeral-sided revision-free survival rate (excluding infection, instability, and glenoid-sided failures): 94% at 5 years; 89% at 10 years; 75% beyond 10 years
Cox et al., 2024 [22]	Instability (5); wound drainage (1)	Proximal graft resorption: 14.3% (not requiring revision) Allograft incorporation: 100%	Revision (6)
Han et al., 2022 [23]	Fracture at allograft-host bone junction (1); dislocation or subluxation (0); local recurrence (0); infection (0)	Bone nonunion of allograft–host junction (0); implant loosening (0); stress shielding (0)	Revision operation (0)
Houdek et al., 2021 [24]	Prosthetic or allograft fracture (2); subluxation (0); infection (0)	Allograft resorption (6, 60%)	Revision procedure (0) Reoperation (18%)
Houdek et al., 2022 [25]	Radial nerve palsy (1); allograft fracture (1); detachment of pectoralis flap (1)	Scapular notching (1); allograft resorption leading to loosening (1, 9%); nonunion (1)	Revision free survival: 2-year: 90%; 5-year: 90%; 10-year: 60%
Lazerges et al., 2017 [26]	Dislocation (1); tumor recurrence (0)	Secondary displacement (0); lysis in the allograft or prosthetic loosening (0); allograft–host junction consolidation (5); nonunion (1); scapular notching (2)	Revision (1)
Levy et al., 2007 [2]	Periprosthetic and polyethylene socket fracture, later dislocation (1); infection (1); dislocation (1)	Glenoid notching (0); graft incorporation (50%)	Revision (1); reoperation other than revision (1)
Sanchez-Sotelo et al., 2017 [27]	Primary group: postoperative hematoma complicated by infection (1) Revision group: dislocation (1); delayed union (1); delayed wound healing (1); allograft fracture (1); periprosthetic fracture (1)	Mean time to union: 7 months (primary: 6 months; revision: 8 months) Incomplete incorporation: 2 in revision group (asymptomatic) Deep infection leading to graft resorption and fragmentation: 1 in primary group (4%) Graft fragmentation: 1 in primary group	Allograft survival rate: 96% at 2 and 5 years Overall survival rate free of implant revision: 92% ± 5% at 2 and 5 years Revision (2); reoperation (4) Overall survival rate free of reoperation for any reason: 80% ± 7% at 2 and 5 years
Zhou et al., 2024 [28]	Periprosthetic fracture (1); local recurrence (0)		
Zuo et al., 2021 [29]	Dislocation (2); infections (0); local recurrence (0); subluxations (0)	Bone union (16); bone resorption (4, 20%); scapular notching (2)	Revision surgeries (2)

## Data Availability

Not applicable.

## Supplementary Materials

The following supporting information can be downloaded at: www.mdpi.com/article/10.3390/jcm13206290/s1, Appendix S1. Search keywords.
