# Supplementary material for: Allograft Prosthetic Composite (APC) for Proximal Humeral Bone Deficiency in Revision Reverse Shoulder Arthroplasty: A Technical Note and Systematic Review
Source: J Clin Med. 2024 Oct 21;13(20):6290. doi: 10.3390/jcm13206290 (PMC11508849; doi:10.3390/jcm13206290)
Supplement: Supplementary file 1 [file jcm-13-06290-s001.zip › jcm-3098102-supplementary.pdf]

Appendix 1. Search keywords.

PubMed/MEDLINE	Results
(((((proximal) AND (humer*)) AND (reverse shoulder)) AND (allograft)) AND (prosthe*)) AND (composite)	n=35
**Embase**	
proximal AND humer* AND ('reverse shoulder' OR (reverse AND ('shoulder'/exp OR shoulder))) AND ('allograft'/exp OR allograft) AND prosthe* AND ('composite'/exp OR composite)	n=33
**Cochrane**	
(allograft prosthesis composite):ti,ab,kw AND (reverse shoulder arthroplasty):ti,ab,kw OR (reverse shoulder replacement):ti,ab,kw"	n=116
